# A mobile health intervention to encourage physical activity in children: a randomised controlled trial

**DOI:** 10.1186/s12887-022-03336-9

**Published:** 2022-05-13

**Authors:** Michelle Ng, Elizabeth Wenden, Leanne Lester, Carri Westgarth, Hayley Christian

**Affiliations:** 1 Telethon Kids Institute, University of Western Australia, Perth, WA Australia; 2 School of Population and Global Health, University of Western Australia, Perth, WA Australia; 3 School of Human Sciences, University of Western Australia, Crawley, WA Australia; 4 Department of Livestock and One Health, Institute of Infection, Veterinary and Ecological Sciences, University of Liverpool, Leahurst Campus, Neston, UK

**Keywords:** Exercise, Children, mHealth

## Abstract

**Background:**

Despite immense benefits of physical activity on health and developmental outcomes, few children achieve recommended daily levels of physical activity. Given more than half of families with children own a dog, we investigated the effect of a mobile health (mHealth) intervention to encourage dog-facilitated physical activity through increased family dog walking and children’s active play with their dog.

**Methods:**

The PLAYCE PAWS study was a three-armed randomised pilot trial conducted in Perth, Western Australia. Children aged 5-10 years with a family dog were randomised to 4 weeks of either 1) SMS-only intervention, 2) ‘SMS + pedometer’ intervention or 3) ‘usual care’ control. The mHealth intervention involved SMS messages to parents; the ‘SMS + pedometer’ group also received a dog pedometer and personalised dog steps diary. Parent-reported measures were collected at baseline, 1- and 3-months post intervention. The primary outcome was weekly frequency of family dog walking and dog play; secondary outcomes were child attachment to the dog and feasibility of the intervention.

**Results:**

A total of 150 children were randomised in staggered blocks to SMS-only (*n* = 50), ‘SMS + pedometer’ (*n* = 50) or usual care (*n* = 50). No differences were observed in family dog walking and dog play at 1-month. SMS-only children (OR 2.6, 95% CI 1.17, 5.83, *P* = 0.019) and all intervention children (OR 1.97, 95% CI 1.01, 3.86, *P* = 0.048) were more likely to increase total dog-facilitated physical activity (sum of family dog walking and dog play responses) at 3-months. The positive associations with total dog-facilitated physical activity disappeared (all *P* > 0.05) after adjusting for socio-demographic factors.

**Conclusions:**

The PLAYCE PAWS mHealth intervention did not significantly affect dog-facilitated physical activity in children. Given high levels of dog ownership in the community, SMS prompts could be a low-cost intervention to encourage more physical activity in children. Further research is needed to understand how increased interaction with the family dog impacts on children’s overall physical activity and other health and development outcomes.

**Trial registration:**

ANZCTR, ACTRN12620000288921, retrospectively registered on 4/3/2020.

## Introduction

Promoting physical activity in childhood is crucial to preventing obesity, and is identified as a priority by the World Health Organization (WHO) [1]. Despite clear known benefits of an active lifestyle, less than half of Australian, Canadian, New Zealand children and about half of UK children meet the recommended 60 minutes of moderate to vigorous physical activity per day [2].

Many families with children own a dog [3–6]; and children with a family dog are more active and likely to meet physical activity recommendations [7, 8]. Children who walk their dog also tend to play in the street and yard and be independently mobile compared with children who don’t walk their dog [9]. Independent mobility (e.g., walking/cycling to school without adult supervision) is an important source of daily physical activity for school-aged children [10, 11].

Despite substantial cross-sectional evidence of the health benefits of dog walking in adults and children, few intervention studies exist. A recent review of 13 studies concluded that dog-facilitated physical activity interventions appear effective, but identified the lack of intervention studies in children [12]. The review also highlighted the potential of dog-facilitated physical activity intervention studies that use mobile health (mHealth) based strategies.

mHealth involves the use of mobile computing and communication technologies in public health [13]. It offers a novel and cost-effective approach to traditional intervention methodologies [14]. mHealth-based physical activity strategies which encourage participant involvement in self-selected physical activities have better longer-term success compared to traditional exercise-based interventions which tend to be closely supervised and expensive [15, 16]. In addition, the WHO supports the use of mHealth strategies to increase physical activity [17].

To date three published studies have utilised mHealth strategies to increase dog-facilitated physical activity. Two studies in U.S. adult dog owners showed an increase in levels of dog walking post intervention. The first study (*n*=102) utilised an online social network to encourage weekly neighbourhood dog walks; participants were also given an activity monitor to track walking [18]. At six months follow-up, both the intervention and control groups increased their daily dog walking. The second study (*n*=49) trialled the use of targeted email messages (twice weekly for 4 weeks, then weekly for 8 weeks) to promote the human and canine benefits of dog walking [19]. Dog walking significantly increased in the intervention group at follow-up, compared with the control group, and the effect was sustained at twelve months. While both studies highlight the merit of mHealth strategies to increase physical activity levels through dog walking, they did not include children and were limited by small sample sizes.

The only intervention study to date which focused on increasing children’s physical activity levels through dog walking and play was the Children, Parents and Pets Exercising Together (CPET) study. The 10-week pilot intervention encouraged children (*n*=28; 9-11 year old) to play/walk with their dog [20], and involved home visits by a qualified animal behaviourist, phone calls and text messages to motivate and review goal progress, and information on dog walking routes and dog play activities. While no significant differences were found between the intervention and control group for physical activity or weekly dog walking, mostly due to the small sample size, families found the intervention to be acceptable and feasible.

Given the high level of family dog ownership and the combined potential of dog-facilitated physical activity and mHealth-based strategies, more dog-facilitated physical activity intervention research involving children is needed [21, 22]. The aim of this study was to determine if a mHealth-based dog-facilitated physical activity intervention increases family dog walking and children’s active play with the family dog.

## Methods

A study protocol for this trial has been published previously [23]. Relevant details are explained below.

### Design and randomisation

PLAYCE PAWS was a three-armed, parallel-group, randomised controlled study conducted in Perth, Western Australia between April 2019 and October 2021. Participants were randomly assigned in staggered blocks to either intervention (SMS-only or ‘SMS + pedometer’) or usual care groups with equal sample sizes in each group (*n* = 50/group; total = 150). Data were collected at baseline, 1- and 3-months post intervention. The study employed an on-going rolling recruitment and implementation of intervention until the target sample size was achieved. The study adhered to the CONSORT guidelines for the design and reporting a randomised trial [24], and the CONSORT checklist is provided as supplementary material. The study was carried out in accordance with the Helsinki Declaration. The experimental protocol (including the parent, child and family dog) was approved by the Human Research Ethics Committee of the University of Western Australia (2021/ET000105 and RA/4/1/7417). The research was conducted in accordance with the Animal Welfare Act (2002) Western Australia and the requirements of the Australian Code of Practice for the Care and Use of Animals for Scientific Purposes (7th Edition 2004). The trial was retrospectively registered with the Australian New Zealand Clinical Trials (ACTRN12620000288921).

### Study sample, recruitment, inclusion and exclusion criteria

**Fig. 1:**
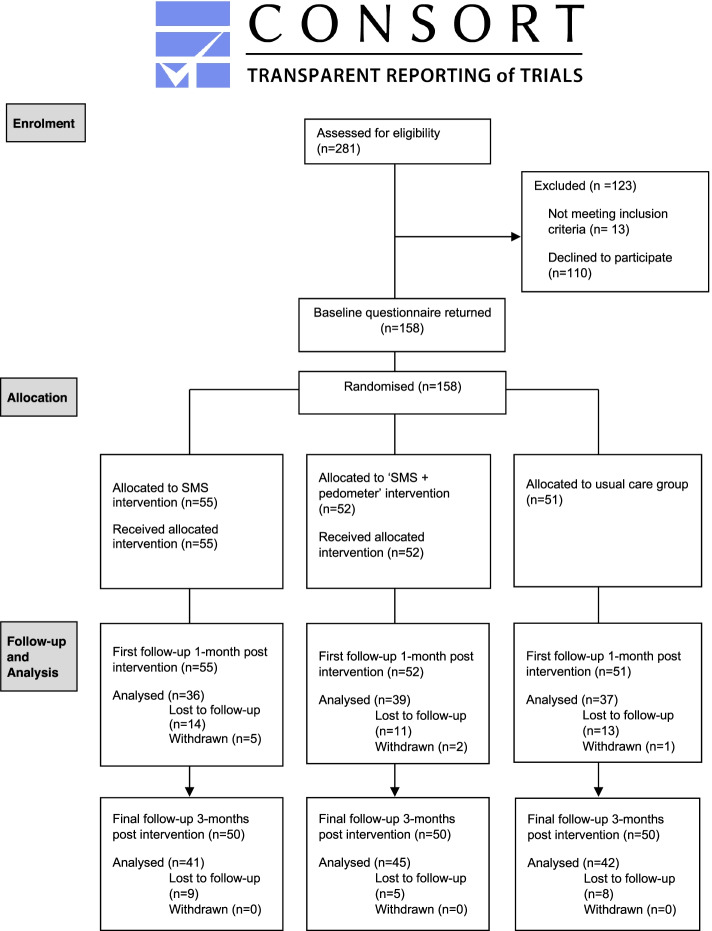
CONSORT flow diagram for randomized trial participants

Participants were recruited from an existing cohort study (PLAYCE) [25] and the general community using multiple strategies including advertising in print (newspapers, school and professional association newsletters) and social media (Facebook and Twitter), crowdsourcing (via institutional websites), market research and through snowball sampling [23]. Parents with children between 5 and 10 years and a family dog(s) that was well socialised with the child and other people were eligible to participate. Children with a recognised disability (physical, emotional/behavioural or intellectual) that affected participation in physical activity were ineligible. Families were included only if they met the inclusion criteria and whose dog passed a dog behaviour screening questionnaire conducted via the telephone by the study team [23]. The screening questions were drafted in consultation with co-author Westgarth, who is a full member of the UK Association of Pet Behaviour Counsellors. Parents provided written informed consent for them and their child’s participation in the study. As the owner of the family dog, parents also provided consent for their family’s dog to participate. Parents were required at all times to supervise interactions between their child and dog to ensure safe dog play and walking practices. This was highlighted in the study information as well as in resources provided to parents on safe interactions between children and the family dog. The CONSORT study flow diagram summarises sample attrition and missing data for outcome measures (Fig. 1).

### Intervention and usual care group

Physical activity-based minimal intervention strategies were tested over a four-week period. Both intervention groups received personalised mHealth SMS message prompts three times a week to motivate and encourage parents to support their child to either walk and/or play with their dog each day. The ‘SMS + pedometer’ group also received a Yamax SW200 pedometer to attach to their dog’s collar and a personalised dog steps diary for children to record the number of pedometer steps their dog did during play or walking. To facilitate family dog walking and dog play, information about dog friendly parks, trails, and beaches, games for children to play with their dog, and tips about how children can safely interact with their dog were also provided. The usual care group continued their normal routine without any contact from researchers for the duration of the study. To ensure fair access to any beneficial outcomes of the project, the usual care group received the same resources at the end of the study.

### Measures

Parents completed three online surveys (baseline, 1- and 3-months post intervention) measuring children’s family dog walking, dog play, level of attachment to the family dog and socio-demographic factors. Full details of the measures have been previously described [23].

#### Children’s family dog walking and dog play

Existing items from the PLAYCE cohort study parent-report survey [25] and adapted from the Healthy Active Preschool Years Study [26] were used to measure children’s weekly frequency of family dog walking and active play with the family dog (response scale: ‘never/rarely’, less than once/week, 1-2 times/week, 3–4 times/week, 5–6 times /week, daily). The reliability of these items is sound (intraclass correlation (ICC) = 0.63) [26]. Total dog-facilitated physical activity was defined as the sum of family dog walking and dog play responses.

#### Dog attachment and socio-demographic factors

The child-dog attachment sub-scale included items from the Dogs and Physical Activity Tool (DAPA Tool) [27] (5-point Likert response scale: strongly disagree to strongly agree). This tool has good to excellent reliability for measuring adult-reported dog attachment levels (ICC= 0.65-0.92) [27]. A previously modified version of The Inclusion of Other in the Self (IOS) Scale [28] (single-item pictorial measure of seven pictures showing overlap of two circles) was used to measure the closeness of the relationship between the child and family dog [29, 30].

Child (sex, age, siblings) and parent (sex, age, highest level of education, work status and family structure) socio-demographic factors were collected using standard items.

### Acceptability of the intervention

As part of the study’s process evaluation, a brief end of study questionnaire was administered to a sub-set of 42 intervention families to understand the acceptability of the intervention strategies and resources and to provide feedback for future interventions. Example of items included: ‘How satisfied were you with the SMS message/dog pedometer/dog steps diary?’ (where applicable); ‘How suitable/motivational/easy to understand were the: SMS message content; information on dog friendly parks, trails and beaches; games for children to play with their dog?’ (5-point Likert response scale: strongly agree to strongly disagree). Open-ended questions such as ideas and feedback for future trials were included.

### Statistical analysis and sample size considerations

Based on PLAYCE study baseline data [25] and CPET study data [31], this study had 80% power to detect a one-unit difference in the pre-post change in the number of times per week children did family dog walking or played with their dog between the intervention and usual care groups (*n* = 50/group). There are no comparable intervention data in this young group of children to accurately inform a power calculation. However, based on PLAYCE study baseline data and CPET study data, it was expected that the response within each group would be normally distributed with a standard deviation of 1.5.

Distributions of key variables were characterised using conventional descriptive statistics, with Chi-square and Mann-Whitney tests used to examine differences in baseline socio-demographic characteristics between groups. Kruskal-Wallis tests were used to examine between group differences at baseline, 1-month and 3-months, and post-hoc analysis was undertaken using the Mann-Whitney test.

Ordinal regression models were performed with family dog walking, dog play and total dog-facilitated physical activity variables. Initial analysis showed no differences between the two intervention groups thus intervention groups were combined to form a ‘combined intervention’ group. First, models were run with outcome measures at 1-month and 3-months (family dog walking, dog play, total dog-facilitated physical activity) as dependent variables, and baseline family dog walking, dog play or total dog-facilitated physical activity and group (SMS-only, ‘SMS + pedometer’, ‘combined intervention’) as independent variables. Subsequent models then adjusted for socio-demographic factors (child age, child sex, parental educational level). The reference group was the usual care group. Analyses were carried out using IBM SPSS Statistics for Windows (Version 26.0; IBM Corp., USA) and statistical significance was set at an alpha of 0.05.

## Results

### Sample characteristics

Table 1 shows baseline characteristics of the participants. Briefly, on average children were 7.3 years of age (SD = 1.2), just over half were boys (56.0%) and most had siblings (74%) Table 1. Ninety percent of parents were women (mean age 40 years, SD 5.7). Most parents had a university degree or higher (66.7%), were in full or part-time employment (86.7%), and in a married/de facto relationship (89.3%). The majority of children felt close (highly attached) to their family dog (88.6%). At baseline, almost 13% of children went on family dog walks five or more times/week and 51% played with their dog daily. There were no socio-demographic differences or differences in dog-facilitated physical activity between groups at baseline.

**Table 1:** Baseline characteristics overall and by group

	Total sample ***n*** = 150 n (%)	SMS group ***n*** = 50 n (%)	‘SMS + pedometer’ group ***n*** = 50 n (%)	Usual care group ***n*** = 50 n (%)	***P*** value (SMS vs usual care)	***P*** value (‘SMS + pedometer’ vs usual care)
***Socio-demographic factors***						
Child mean age (SD)	7.30 (1.22)	7.42 (0.93)	7.07 (1.30)	6.87 (1.06)	0.43	0.43
Child sex (boys)	84 (56.0)	29 (58.0)	32 (64.0)	23 (46.0)	0.32	0.05
Parent mean age (SD)	40.05 (5.68)	40.47 (5.24)	39.56 (5.34)	39.1 (8.52)	0.32	0.83
Parent sex (female)	135 (90)	49 (98.0)	41 (82.0)	45 (90.0)	0.20	0.19
Parent education						
* Secondary level*	19 (12.7)	6 (12.0)	9 (18.3)	4 (8.0)	0.52	0.42
* Trade/diploma*	34 (22.6)	15 (30.0)	9 (18.4)	10 (20.0)		
* University/Post-graduate*	97 (66.7)	30 (52.0)	31 (63.2)	36 (72.0)		
Work status						
* Full-time*	60 (40.0)	20 (40.0)	16 (32.0)	24 (48.0)	0.88	0.44
* Part-time*	70 (46.7)	24 (48.0)	27 (54.0)	19 (38.0)		
* Not working*	3 (2)	2 (4.0)	0 (0.0)	1 (2.0)		
* Home duties*	17 (11.3)	4 (8.0)	7 (14.0)	6 (12.0)		
Family structure						
* Partnered*	134 (89.3)	43 (86.0)	44 (88.0)	47 (94.0)	0.51	0.66
* Single parent*	16 (10.7)	7 (14.0)	6 (12.0)	3 (6.0)		
Siblings	111 (74.0)	39 (78.0)	37 (74.0)	35 (70.0)	0.66	0.74
***Child attachment to dog***						
Level of attachment to dog (SD)^a^	4.39 (0.68)	4.40 (0.67)	4.34 (0.82)	4.42 (0.52)	0.62	0.86
Feeling of closeness to dog^b^						
* Less overlap*	17 (11.4)	4 (8.0)	5 (10.0)	8 (16.3)	0.68	0.35
* More overlap*	133 (88.6)	46 (92)	45 (90.0)	42 (83.9)		
***Dog-facilitated physical activity (with child)***						
Family dog walking						
* Less than once / week*	52 (32.7)	17 (34.0)	14 (28.0)	17 (34.7)	0.35	0.24
* 1 -2 times / week*	49 (30.8)	14 (28.0)	15 (30.0)	18 (36.7)		
* 3 -4 times / week*	38 (23.9)	12 (24.0)	13 (26.0)	12 (24.5)		
* ≥ 5 times / week*	20 (12.6)	7 (14.0)	8 (16.0)	2 (4.1)		
Active play with family dog						
* ≤ 2 times /week*	28 (17.5)	9 (18.0)	5 (10.0)	13 (26.5)	0.54	0.10
* 3 – 6 times / week*	49 (30.6)	14 (28.0)	17 (34.0)	14 (28.6)		
* Daily*	82 (51.2)	27 (54.0)	28 (56.0)	22 (44.9)		

^a^6-item measure of pet attachment, scale 1 to 7, higher scores represent greater the attachment to the dog

^b^Inclusion of Other in the Self Scale, a visual scale using two circles (one for child and one for dog) with differential overlap. The greater the overlap the closer the relationship with the other

### Change in family dog walking and active play with dog

**Fig. 2:**
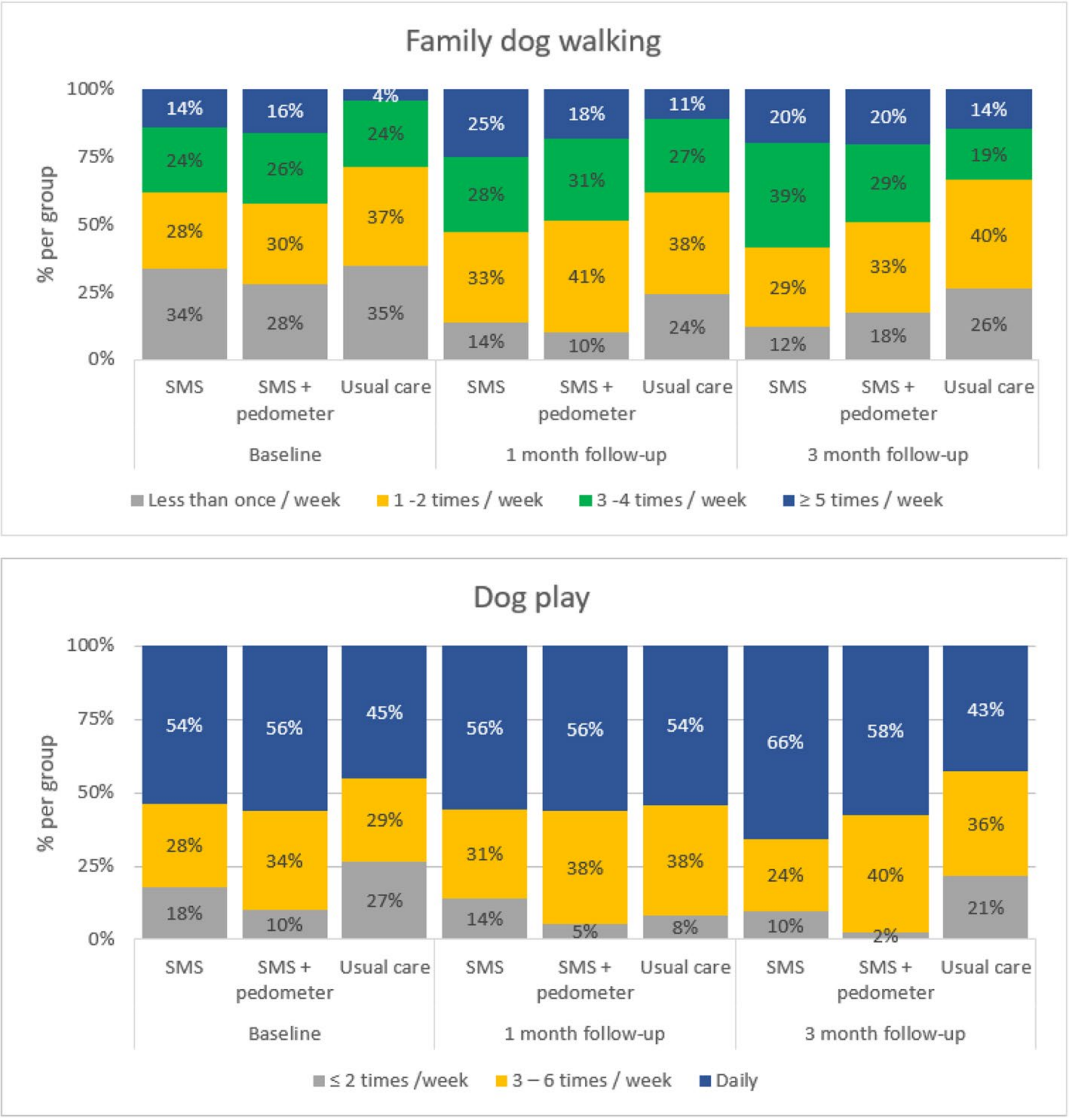
Change in family dog walking and dog play between SMS, ‘SMS + pedometer’ and usual care groups at baseline, 1-month and 3-months follow-up

A higher proportion of children in both intervention groups walked their dogs more than five times a week at 1-month compared with the usual care group, and the changes were sustained at 3-months (Fig. 2). A lower proportion of children in both intervention groups walked their dog less than once a week at both follow-up timepoints compared with the usual care group. No differences were statistically significant.

A higher proportion of children in both intervention groups played with their dog daily at 1-month compared to the usual care group, and the changes were sustained at 3-months (Fig. 2). A lower proportion of both intervention groups played with their dog less than twice a week at 3-months compared with the usual care group. No differences were statistically significant.

### Adjusted change in family dog walking, dog play and total dog-facilitated physical activity between groups

The SMS-only group were 2.6 times significantly more likely to increase their frequency of total dog-facilitated physical activity at 3-months, compared with the usual care group, after adjusting for baseline total dog-facilitated physical activity levels (OR 2.6; 95% CI 1.17, 5.83) Table 2. After further adjusting for socio-demographic factors, the findings only remained significant at *p*<0.1. Similar positive changes in the SMS-only group’s family dog walking at 1-month and 3-months and dog play at 3-months approached significance (0.05<*p*<0.1), after adjusting for baseline family dog walking or dog play levels respectively. For the ‘SMS + pedometer’ group, there were no significant differences in family dog walking, dog play or total dog-facilitated physical activity at 1-month and 3-months. The combined intervention group were two times significantly more likely to increase their frequency of total dog-facilitated physical activity compared with the usual care group at 3-months, after adjusting for baseline total dog-facilitated physical activity levels (OR 1.97; 95%CI: 1.01, 3.86). After further adjusting for socio-demographic factors, the findings only remained significant at *p*<0.1. Positive changes in the ‘combined intervention’ group’s dog play at 3-months approached significance (0.05<*p*<0.10), after adjusting for baseline dog play levels only.

**Table 2:** Adjusted associations between groups and family dog walking, dog play and dog-facilitated physical activity

	Model 1				Model 2			
	Baseline to 1-month follow-up		Baseline to 3-months follow-up		Baseline to 1-month follow-up		Baseline to 3-months follow-up	
	OR (95%CI)	***P*** value	OR (95%CI)	***P*** value	OR (95%CI)	***P*** value	OR (95%CI)	***P*** value
***SMS group***^**a**^								
Family dog walking	2.15 (0.89, 5.21)	0.09*	2.20 (0.97, 5.04)	0.061*	1.60 (0.62, 4.18)	0.33	1.82 (0.73, 4.54)	0.20
Dog play	0.87 (0.32, 2.35)	0.78	2.47 (0.97, 6.29)	0.058*	0.79 (0.28, 2.25)	0.67	1.89 (0.72, 4.98)	0.20
Total dog-facilitated physical activity	1.70 (0.73, 3.97)	0.22	2.61 (1.17, 5.83)	0.019**	1.51 (0.60, 3.81)	0.38	2.20 (0.93, 5.21)	0.072*
***‘SMS + pedometer’ group***^**b**^								
Family dog walking	1.63 (0.68, 3.88)	0.27	1.18 (0.54, 2.57)	0.68	1.59 (0.64, 3.96)	0.32	1.29 (0.57, 2.94)	0.54
Dog play	0.63 (0.23, 1.73)	0.37	1.70 (0.73, 3.96)	0.22	0.49 (0.17, 1.43)	0.19	1.71 (0.70, 4.17)	0.24
Total dog-facilitated physical activity	1.18 (0.52, 2.68)	0.69	1.54 (0.71, 3.35)	0.27	1.09 (0.46, 2.55)	0.85	1.67 (0.75, 3.71)	0.21
***‘Combined intervention’ group***^**c**^								
Family dog walking	1.83 (0.87, 3.85)	0.11	1.60 (0.81, 3.16)	0.18	1.56 (0.71, 3.39)	0.27	1.63 (0.79, 3.33)	0.19
Dog play	0.72 (0.29, 1.74)	0.46	2.02 (0.94, 4.35)	0.072*	0.60 (0.24, 1.51)	0.28	1.84 (0.83, 4.06)	0.13
Total dog-facilitated physical activity	1.38 (0.67, 2.82)	0.38	1.97 (1.01, 3.86)	0.048**	1.16 (0.55, 2.46)	0.70	1.98 (0.98, 3.97)	0.056*

^a^SMS group (*n*= 50) compared to usual care group (*n*=50). ^b^‘SMS + pedometer’ group (*n*=50) compared to usual care group (*n*=50). ^c^’Combined intervention’ group (*n*=100) compared with usual care group (*n*=50). Model 1 adjusted for baseline family dog walking, dog play or total dog-facilitated PA as relevant. Model 2 adjusted for factors in Model 1 and child sex, child age and parental educational level. Ref = usual care group. *p<0.1; ** *p*<0.05

### Intervention feasibility and acceptability

Most parents found the intervention strategies acceptable with 81% satisfied to very satisfied with the SMS prompts, 83% satisfied to very satisfied with the dog pedometer, and 67% satisfied to very satisfied with the dog steps diary.

Parents indicated that the SMS messages were motivating and provided prompts to get active: “Good to get a reminder to keep on going with the efforts to exercise and play with the dog”; “Reminded me of what I could be doing with the dog and kids”. Areas for improvement included increasing the frequency of SMS messages, to schedule messages in mid-afternoon periods around the time when parents collect children from school and having online alternatives for completing the dog steps diary.

## Discussion

There were no between-group differences in dog-facilitated physical activity at 3-months, however borderline associations were observed between dog-facilitated physical activity for SMS and ‘combined intervention’ groups at 3-months. Our findings suggest that a simple mHealth intervention may encourage children to spend more time being physically active with their dog.

To the best of our knowledge, this is one of the first studies that employs a mHealth dog-facilitated intervention to increase children’s physical activity. In support of our findings, reviews of children’s physical activity interventions show most interventions have a small to negligible-small effect on children’s physical activity levels [32–34]. Similarly, a review of electronic-based interventions on children’s physical activity reported that interventions have positive but short-lived effects [35]. Further research with larger sample sizes is needed to better understand the effectiveness of dog-facilitated physical activity interventions on children’s dog-facilitated physical activity and overall physical activity levels.

The dog pedometer (and dog steps diary) did not appear to have any additional effect on children’s dog-facilitated physical activity, over and above the effect of the SMS prompts. SMS prompts were the main mHealth strategy used in this study; however a dog pedometer was trialled as an additional tool to encourage children to interact and be more physically active with their dog. Anecdotal evidence indicated that both parents and children thought the dog pedometer was novel and fun, and children enjoyed completing the dog steps diary. Pedometers have been previously used with success in adult population physical activity campaigns such as promoting 10,000 steps per day [36], or doing 3000 steps in 30 minutes [37]. As such, future studies could consider incorporating the use of pedometers with personalised step counts diaries to support children’s involvement. However, further research is needed to understand the value of dog pedometers as an intervention strategy as well as its use as an objective proxy measure of change in children’s physical activity levels.

Significant increases in total dog-facilitated physical activity were observed at 3-months rather than 1-month follow-up. This is in line with habit formation research which suggests that it takes 18 to 265 days to form a habit [38]. As physical activity behaviour is complex and multidimensional [39], a longer time may be required to change behaviour and for a new habit to be formed. Similar findings were reported by Richards et al. who found that significant increases in family dog walking in the intervention group only occurred at 6 months and were also sustained at 12 months [19]. A longer intervention and follow-up period would enable further investigation of the time taken for family dog walking and dog play behaviours to change and to be maintained, as well as the factors influencing dog-facilitated physical activity becoming habit or routine in families.

At baseline approximately 60% of families walked their dogs more than once a week, and this increased to more than 75% at follow-up. In contrast, similar aged children (5-6 years and 10-12 years) in an Australian cross-sectional study engaged in family dog walking at most once or twice a month (parent-report) [5]. However, children in the CPET study had similar dog walking frequencies as the current study - children walked with their dogs 2-3 times a week [31]. Similarly in the current study, more than 80% of intervention children played with their dog three or more times a week across all time points. Data from another Australian study reported 70% of primary school-aged (5-12 years) children played with their dog at least once in the past week [7]. These findings highlight the need for further epidemiological studies to establish population levels of dog-facilitated physical activity in children. This could be added as single items to routinely collected national health and wellbeing surveys.

Parents play an integral role in supporting and managing young children’s physical activity opportunities [40, 41]. Yet, parent and family busy lifestyles are a barrier to children’s unstructured physical activity including family dog walking and dog play [42]. However, it has been suggested that dogs may alleviate children’s and/or their parents’ concerns about neighbourhood safety by providing a sense of protection from personal harm [43]. Future studies could investigate if dog-facilitated physical activity interventions impact parent and child perceptions of safety and facilitate more family dog walking and child independent mobility.

The PAWS mHealth intervention has potential to be scaled up and implemented in community wide physical activity programs. The feasibility results indicated that the PAWS intervention was acceptable and reasonable with a study retention rate (90%) comparable to other physical activity intervention studies [31, 44, 45]. As a large proportion of the population - particularly households with children, have a dog, the potential impact of dog-facilitated physical activity interventions for increasing children’s (and other family member’s) physical activity levels is significant. Similar calls have been made by Rhodes et al. in their review of dog-facilitated physical activity interventions [12]. For example, public health messaging could highlight how easy it is for existing dog-owning families to be active with the family dog. In addition, the value of walking and playing with the family dog for not only the child and dog but also for parents and other carers should be emphasised. Future studies should investigate the impact of dog-facilitated physical activity interventions on parents’ and other family members physical activity levels as well as trial alternative mHealth strategies such paid social media advertising.

Our study had several limitations. First, the sample size albeit larger than most dog-facilitated physical activity interventions to date, could have reduced the ability to detect significant changes in children’s dog-facilitated physical activity. Second, the findings were based on parent-report data which may have introduced response biases such as social desirability bias. Also, outcome measures for dog walking and dog play lacked some sensitivity, highlighting the need for future studies to collect information on the duration and frequency of dog-facilitated physical activity as well as objective measures such as accelerometry. Finally, variation in weather conditions during the study may have influenced parent’s decisions to go on family dog walks. However, previous studies suggest that the weather including the time of year/season has minimal impact on dog walking behaviour [46–48]. Furthermore, the study site (Perth, Western Australia) has a Mediterranean climate with relatively little variation in weather conditions across all seasons [49]. Study strengths include the high study retention rate, context-specific outcome measures, child attachment to the family dog measure and two follow-up timepoints.

## Conclusions

In summary, this is one of the first, to our knowledge, intervention of a mHealth dog-facilitated strategy to increase children’s physical activity. The mHealth intervention did not increase children’s family dog walking or dog play, after adjusting for socio-demographic factors. Further studies are needed confirm or contrast our results. Despite a high proportion of dog-owning families in the community, many children do not gain the physical activity benefits of walking and actively playing with the family dog. Interventions, policies and community programs should capitalise on the high level of dog ownership and incorporate dog walking or dog play messaging into broader mass media physical activity campaigns. SMS prompts could be one way to disseminate physical activity messages within the community.

## Data Availability

The datasets used and/or to be analysed during the current study, in addition to data collection forms are available from the corresponding author on reasonable request.
